# Strategies for specialty training of healthcare professionals in low-resource settings: a systematic review on evidence from stroke care

**DOI:** 10.1186/s12909-023-04431-w

**Published:** 2023-06-16

**Authors:** Junaid Habibi, Jackie Bosch, Patrick Bidulka, Sarah Belson, Vincent DePaul, Dorcas Gandhi, Anne Kumurenzi, Rita Melifonwu, Jeyaraj Pandian, Peter Langhorne, John M. Solomon, Dimple Dawar, Sandra Carroll, Gerard Urimubenshi, Martin Kaddumukasa, Leah Hamilton

**Affiliations:** 1 Michael G. DeGroote School of Medicine, McMaster University, Hamilton, ON Canada; 2 Population Health Research Institute, Hamilton, ON Canada; 3 School of Rehabilitation Science, McMaster University, Hamilton, ON Canada; 4 London School of Hygiene and Tropical Medicine, London, England, UK; 5 Stroke Association, London, England, UK; 6 School of Rehabilitation Therapy, Queen’s University, Kingston, ON Canada; 7 Department of Neurology, Christian Medical College, Ludhiana, Punjab India; 8 Stroke Action Nigeria, Onitsha, Nigeria; 9 Institute of Cardiovascular & Medical Sciences, University of Glasgow, Glasgow, Scotland, UK; 10 Department of Physiotherapy, Manipal Academy of Higher Education, Manipal, Karnataka India; 11 School of Nursing, McMaster University, Hamilton, ON Canada; 12 College of Medicine and Health Sciences, University of Rwanda, Kigali, Rwanda; 13 Department of Medicine, School of Medicine, College of Health Sciences, Makerere University, Kampala, Uganda

**Keywords:** Low-resource, Stroke, Training, Education, Teaching, Telehealth, Multidisciplinary, Task-shifting, Train-the-trainer, Workshop

## Abstract

**Background:**

The greatest mortality and disability from stroke occurs in low- and middle-income countries. A significant barrier to implementation of best stroke care practices in these settings is limited availability of specialized healthcare training. We conducted a systematic review to determine the most effective methods for the provision of speciality stroke care education for hospital-based healthcare professionals in low-resource settings.

**Methods:**

We followed the PRISMA guidelines for systematic reviews and searched PubMed, Web of Science and Scopus for original clinical research articles that described or evaluated stroke care education for hospital-based healthcare professionals in low-resource settings. Two reviewers screened titles/abstracts and then full text articles. Three reviewers critically appraised the articles selected for inclusion.

**Results:**

A total of 1,182 articles were identified and eight were eligible for inclusion in this review; three were randomized controlled trials, four were non-randomized studies, and one was a descriptive study. Most studies used several approaches to education. A “train-the-trainer” approach to education was found to have the most positive clinical outcomes (lower overall complications, lengths of stay in hospital, and clinical vascular events). When used for quality improvement, the “train-the-trainer” approach increased patient reception of eligible performance measures. When technology was used to provide stroke education there was an increased frequency in diagnosis of stroke and use of antithrombotic treatment, reduced door-to-needle times, and increased support for decision making in medication prescription was reported. Task-shifting workshops for non-neurologists improved knowledge of stroke and patient care. Multidimensional education demonstrated an overall care quality improvement and increased prescriptions for evidence-based therapies, although, there were no significant differences in secondary prevention efforts, stroke reoccurrence or mortality rates.

**Conclusions:**

The “train the trainer” approach is likely the most effective strategy for specialist stroke education, while technology is also useful if resources are available to support its development and use. If resources are limited, basic knowledge education should be considered at a minimum and multidimensional training may not be as beneficial. Research into communities of practice, led by those in similar settings, may be helpful to develop educational initiatives with relevance to local contexts.

**Supplementary Information:**

The online version contains supplementary material available at 10.1186/s12909-023-04431-w.

## Background

Stroke is the second leading cause of death worldwide [1], and an estimated 86% of stroke deaths occur in low- and middle-income countries [2]. Rates of disability and mortality from stroke are 10 times greater in medically underserved regions in comparison to the world’s most developed nations [3], and the implementation of best practices in stroke care is especially difficult [4]. In some areas, hospital-based healthcare professionals have low knowledge of stroke and its care [5, 6], and according to the World Stroke Organization, the level of training for healthcare professionals varies, with formal training unavailable in many regions [7]. Many barriers exist for healthcare professionals to obtain formal stroke care training, including lack of financial support for training, health policies and government priorities that may not include specialty stroke care training, limited training opportunities that are relevant to the culture and context, and a lack of evidence on the effectiveness of interventions in low-resource settings and subsequently the lacking existence of guidelines [8]. To address these barriers, there is a significant need for the development of evidence-based context-specific approaches to stroke care education for healthcare professionals in low-resource settings.

To better understand the optimal methods for providing training in low-resource settings, we searched for studies that addressed our research question “what are the optimal methods for educating hospital-based healthcare professionals in low-resource settings on stroke care”. We sought to describe and compare the reported impact of these interventions on knowledge, behaviours and outcomes in these environments. To our knowledge, no study has systematically analyzed primary literature to determine an optimal approach to stroke training in low-resource settings. This systematic review was conducted to provide clinicians and policy makers evidence and examples to aid in the development and implementation of specialty stroke care education for hospital-based healthcare professionals in low-resource settings.

## Methods

This review was conducted according to the guidelines of the Preferred Reporting Items for Systematic Reviews and Meta-Analyses (PRISMA) systematic review approach [9].

### Eligibility criteria

Original clinical research articles (descriptive or evaluative) were eligible if they described or evaluated stroke care education (any intervention or model for the treatment of stroke patients) for hospital-based healthcare professionals in low-resource settings. Healthcare professionals were defined to be any credentialed health professional (those with degrees or diplomas) working in the hospital (recognized to be a hospital employee with an official role) involved in stroke care, excluding patients, family, and other caregivers. Hospitals were defined as any healthcare facility, inpatient or outpatient, in which health professionals were present.

### Search strategy and study selection

A systematic search of the literature was conducted using the following databases: PubMed, Web of Science and Scopus. Search terms included both MeSH terms and free text. The search strategy is available in Additional file 1.

Articles were imported into Covidence [10] on February 3^rd^, 2021. Covidence was used for the independent screening of titles and abstracts together. All study designs were included, and references lists were hand searched for primary studies that may have been missed. Title and abstract screening were conducted by two reviewers (JH/LH). Articles describing original study results were eligible for full review if the study met the eligibility criteria. If consensus was not reached at title and abstract screening, then full text screening took place. If at full text screening there was a lack of consensus, a third senior reviewer (JB) made the final decision on inclusion.

### Data extraction

The information extracted from each study included the lead author, year of publication, country of study, study design, participants/sample size, healthcare site characteristics, descriptions of educational strategies, description of educational aims, the outcome measures, overall results, and comparisons included in studies. In addition to describing the characteristics of each study we used the Kirkpatrick model, a commonly used approach to evaluate training programs [11, 12]. The model uses four levels of training outcomes: reaction, learning, behaviour and results to evaluate training.

### Assessment of methodological quality

The methodological quality of the included studies was assessed by JH, SB, PB and VD using the Joanna Briggs Institute/University of Adelaide critical appraisal tools [13].

### Identification of themes

Due to the heterogenous nature of the included literature, a meta-analysis was not possible. As such, the identified educational interventions were grouped thematically, based on similar characteristics and objectives, and narratively described.

## Results

**Fig. 1:**
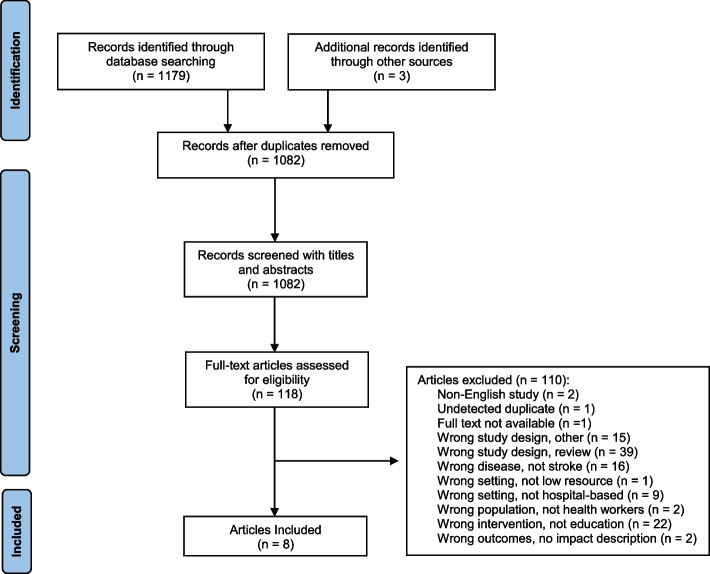
Flow chart for study screening and selection using PRISMA methodology. Preferred Reporting Items for Systematic Reviews and Meta-Analyses (PRISMA) guidelines for systematic reviews were followed in the screening of studies for inclusion in this systematic review. Covidence was used to screen studies gathered from databases. Presented reasons for study exclusion in full-text screening were ordered by JH and LH in order of perceived importance. A flow chart template was derived from the guidelines of the PRISMA organization and was modified with study screening data from Covidence

After removal of duplicate records, 1082 records were screened and 964 were removed primarily because they did not describe educational approaches, were not specific to stroke training or did not occur in low-resources settings (Fig. 1). A total of 118 articles went forward for full review, from which 110 were excluded, because they were: not original clinical research articles (49%), did not include any educational component in the intervention (20%), or did not discuss stroke (15%).

### Study characteristics

Eight articles describing eight educational interventions were eligible for inclusion in this systematic review (see Fig. 1) [14–21]. Characteristics of the included studies and the educational interventions are available in Table 1. In summary, we identified three randomized controlled trials [16, 18, 19], one case series [20], two pre-post studies [15, 17], one survey [21], and one descriptive study [14]. Studies were conducted in Argentina [19], Brazil [15, 19], China [16, 18], Ghana [14], India [17, 20], Nigeria [21] and Peru [19] and all except one were in an outpatient setting.

**Table 1:** Characteristics of included studies

Lead Author (Year), Country	Study Designs, Descriptive/Evaluative	Themes	Participants/Sample Sizes	Healthcare Site Characteristics	Description of Educational Strategies	Included Comparisons/Outcomes	Kirkpatrick Evaluation model level(s)
Carvalho Jr (2019), Brazil [15]	Retrospective pre-/-post, Evaluative	Use of Technology to Provide Education for Stroke Care	Professionals: - 122 attendees Patients: - 90 patients pre-intervention - 199 patients post-intervention	1 “hub” facility: - Neurologists - Remote access of spoke facility desktops for assessment of radiological images 5 “spoke” community EDs with: - Laboratories - Computed tomography (CT) scanners - Telemedicine technologies - One with magnetic resonance imaging machine	- “Hub” facility neurologists provided training to all emergency department staff in the “spoke” facilities - Brief lectures - Live simulations - Encouragement for teamwork	Included comparisons (evaluative): - Number of stroke diagnoses - Door-to-needle time - TBL rates - Symptomatic intracranial hemorrhage rates	Level 4- Results Study reported on stroke outcomes pre-post and overall system usage
Sharma (2016), India [20]	Retrospective pre-/-post case series, Evaluative	Use of Technology to Provide Education for Stroke Care	Professionals: - 4 neurologists - Medical officers (medicine graduates and internal medicine postgraduates), total unknown Patients: - Total unknown - 22 patients received TBL	2 tertiary “hub” facilities with: - Neurologists - “WhatsApp” for radiological image examinations and consultations 17 “spoke” government-run district hospitals: - CT scan facilities - Tissue plasminogen activator - “WhatsApp” to share radiological images	- Medical officers from district hospitals attended workshops on stroke recognition and CT scans - Thrombolysis protocols in ischemic stroke were provided	Included comparisons (quantitative): - Number of patients that received TBL	Level 4- Results Study reported on stroke outcomes pre-post
Wu (2019), China [16]	Cluster randomized control trial, Evaluative	Use of Technology to Provide Education for Stroke Care	Professionals: - Village doctors - Township hospital healthcare providers - County managers - Stroke specialists (app design consultations)	- Each village had a village doctor - 50 villages, 25 villages in intervention arm	- Education was delivered through a mobile health (mHealth) application designed by the research team - The application included videos, graphs, texts, and a list of important medications to assist village doctors’ decision making on medication prescription	Included information from feasibility survey results at 12 months of the main trial (quantitative): - Village doctor’s ratings on a Likert scale with questions regarding ease and speed of learning how to use app, app’s aid in prescribing medication, and enthusiasm for continued app usage	Level 1- Reaction Village doctors satisfaction with the mHealth application
Akinyemi (2015), Nigeria [21]	Pre-/-post survey, Evaluative	On-Site Workshops for Education on Task- Shifting to Develop Knowledge on Stroke for Non-Specialists	Professionals: - 210 non-neurologist health workers (medical officers, nurses, and community health extension workers)	- Two federal tertiary healthcare centers	- One-day basic stroke course - Didactic presentations - Educational stroke documentary film - Practical/interactive sessions on cases	Included comparisons (quantitative): - Knowledge of risk factors, stroke symptoms, development of stroke, swallowing tests, and use of thrombolytics	Level 2- Learning Pre-post training questionnaire on stroke knowledge
John (2021), India [17]	Pre-/-post cohort study, Evaluative	Training the Trainer for the Implementation of Multidisciplinary Stroke Units	Professionals: - 1 internal medicine physician, 126 nurses, 2 physiotherapists, and 1 occupational therapist Patients: - 125 before stroke unit (pre-SU) - 125 after stroke unit (post-SU) - Both groups age and gender matched	Secondary care hospital with: - CT scanner - Six SU beds - No neurologist	- Physician was trained first over Skype for 2-h sessions that occurred three times a week, over the course of a month - Physician then trained other professionals in 2-h sessions that were conducted for 5 days a week, over 1 month - Stroke unit manual was developed and written by the research group - Multidisciplinary teams were developed to meet once a week	Included comparisons (quantitative): - Overall incidence of complications - Lengths of hospital stay - Rates of swallowing assessment, mobility assessment and patient re-education - Observed functional outcomes after 1-month follow up, as recorded by using the modified Rankin scale	Level 4- Results Study reported on stroke outcomes pre-post stroke unit training
Johnson (2017), Ghana [14]	Descriptive	Training the Trainer for the Implementation of Multidisciplinary Stroke Units	Professionals: - Multidisciplinary team of 3 physiotherapists, 1 occupational therapist, 1 speech language therapist, and 1 stroke physician from the Southwest of England - Professionals from the hospital	Korle Bu Teaching Hospital Accra	- Workshops on the clinical aspects of stroke management included presentations and small group discussions - Leaders from medicine, nursing, and physiotherapy were identified and trained in clinical knowledge, skills, and developing leadership qualities - Deputy leaders were also appointed to work with the clinical leaders and for succession planning - These leaders took responsibility for leading their respective services and educating their professional groups on core skills - The train-the-train approach was supported with teaching materials and a practical competency framework	Outcomes included (qualitative): - Results from implementing the “train the trainer” model for creating a multidisciplinary SU	not applicable
Wang (2018), China [18]	Randomized clinical trial, Evaluative	Training the Trainer for Quality Improvement	Professionals: - Personnel that took care of acute ischemic stroke (AIS) patients Patients: - 4800 patients hospitalized with AIS, 2400 in intervention group and 2400 in control group - Mean number of patients in each cluster was 120 (range:102–145) - 3980 (82.9%) patients at 12-month follow-up, 2003 (83.5%) in intervention group and 1977 (82.4%) in control group	Inclusion criteria: - Secondary or tertiary centre - EDs - Neurological wards - Capacity to administer intravenous r tPA Characteristics: - Intervention and control groups composed of 20 hospitals each - 72.5% were tertiary hospitals - 62.5% had a stroke unit - 62.5% were teaching hospitals - Median annual number of beds of neurological wards was 77 (IQR: 61–178)	- Neurology department director and physician or nurse from each site attended a 2-day workshop - These professionals were then tasked with sharing operational methods with personnel from their sites - An evidence-based clinical pathway written by a panel of stroke experts was provided - Written care protocols for implementation of performance measures were provided - Full-time quality coordinator at each site and monitoring and feedback system for performance measures was implemented	Included comparisons (quantitative): - Patients receiving eligible performance measures (assessed as a whole and as all-or-none measure) - New clinical vascular events at 3, 6, and 12 months	Level—3 Behaviour Level—4 Results Adherence to stroke performance measures for hospital personnel and patient outcomes
Machline-Carrion (2019), Brazil, Argentina, and Peru [19]	Cluster randomized clinical trial, Descriptive	Multidimensional Education for Quality Improvement	Patients: - Diagnosed with AIS or transient ischemic attack (TIA) Hospitals: - 19 hospitals randomized to intervention (817 patients in primary analysis until discharge, 684 at 90 days follow-up) - 17 hospitals randomized to routine care (807 patients in primary analysis until discharge, 698 after 90 days follow-up) Professionals: - Physicians - Nurses	Inclusion Criteria: - 24-h emergency care, with at least 1 physician in charge for the unit for 24 h - 1 on-call neurologist - Central nervous system (CNS) imaging and alteplase therapy available Characteristics: - 47.2% stroke units - 88.9% intra-arterial TBL capabilities 24 h a day - 77.8% teaching hospitals - Median ED volume 1500 patients/month (IQR: 500–3700)	- 2 healthcare professionals from each hospital attended a simulation-based workshop on implementing the intervention - On-site training visits for training healthcare professionals on their hospital’s clinical pathway - Web and telephone training - Audit and feedback reports - Roadmap with therapeutic plan - Checklists for following therapeutic plan - Educational posters - Video on training techniques	Included comparisons (quantitative): - Prescriptions for evidence-based therapies - Secondary prevention efforts - Stroke reoccurrence - Total mortality - Cardiovascular mortality - Hemorrhagic transformations	Level—3 Behaviour Level—4 Results Adherence to stroke performance measures for hospital personnel and patient outcomes

### Critical appraisal

**Fig. 2:**
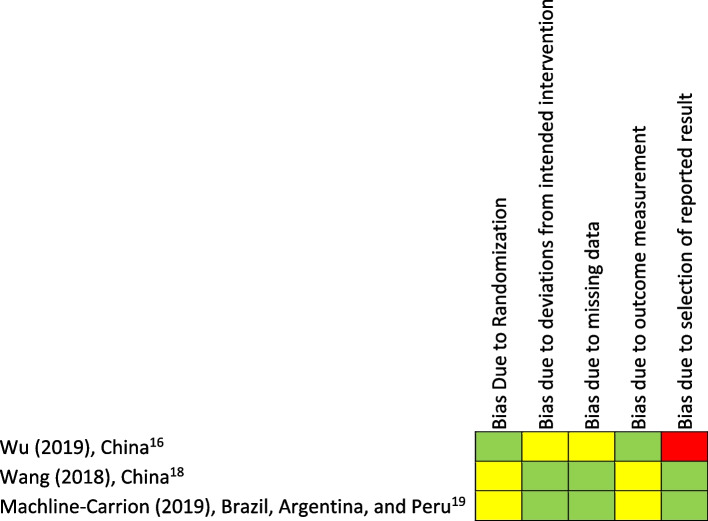
Critical appraisal of randomized studies. The methodological quality of the included studies was determined by JH after consideration of assessments done by JH, SB, PB and VD, using the Joanna Briggs Institute/University of Adelaide critical appraisal tools. Study first authors, years of publication, and country of origin are listed vertically, and different biases are listed horizontally. A “traffic light” model was used to describe concerns for various biases. Low, some, and high concerns for biases were indicated with green, yellow, and red colours respectively

**Fig. 3:**
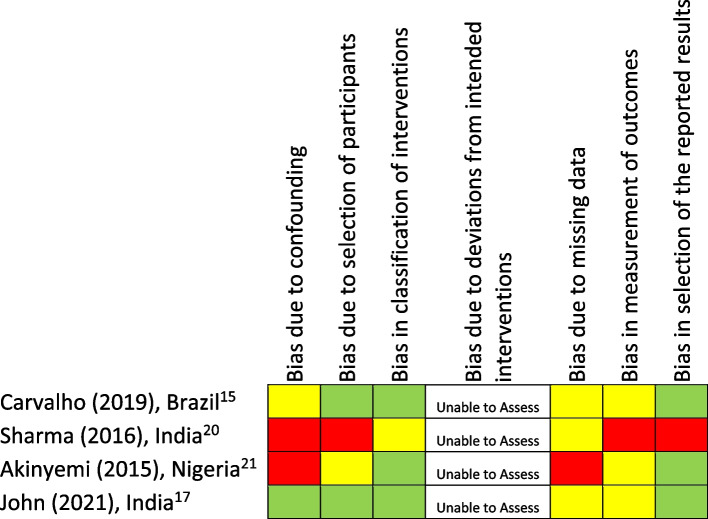
Critical appraisal of non-randomized studies. The methodological quality of the included studies was determined by JH after consideration of assessments done by JH, SB, PB and VD, using the Joanna Briggs Institute/University of Adelaide critical appraisal tools. Study first authors, years of publication, and country of origin are listed vertically, and different biases are listed horizontally. A “traffic light” model was used to describe concerns for various biases. Low, some, and high concerns for biases were indicated with green, yellow, and red colours respectively

A summary of the critical appraisal for randomized controlled trials are presented in Fig. 2 and for non-randomized studies in Fig. 3. Most studies were of satisfactory quality of evidence. However, there were some concerns. Specifically, the study by Sharma et al. was missing the total number of patients that were eligible for thrombolysis [20], the study by Akinyemi et al. had relatively low pre-intervention survey completion at 55.2%, [21] and the study by Johnson et al. [14] was not evaluated for quality as it was descriptive in nature. Five studies could not be assessed for the ‘bias due to deviations from intended interventions’ category as no pre-study protocols were available.

### Methods of education delivery

The studies used a variety of methods for delivery of education. The majority of studies (*n* = 7) utilized more than one educational strategy [14, 15, 17–21]. On-site training was used in seven studies [14, 15, 17–21]. Within these studies, didactic lectures were used in two studies [15, 21], workshops and interactive sessions were used in five studies [14, 18–21], and live simulations were provided in two studies [15, 19]. Written materials for education such as guidelines, manuals, and roadmaps were provided in four studies [14, 17–19]. Videos and virtual training were both provided in two studies [16, 19]. Details on the educational methods used for each study are provided in Table 1.

### Overview of themes in stroke-education related literature

Five themes emerged from the included interventions – 1) use of technology to provide education for stroke care, 2) on-site workshops for task-shifting education to develop knowledge on stroke for non-specialists, 3) “training-the-trainer” for the implementation of multidisciplinary stroke units, 4) “training-the-trainer” for quality improvement, and 5) multidimensional education for quality improvement, when multiple educational themes were present.

### Use of technology to provide education for stroke care

Three studies [15, 16, 20] described interventions designed to support the implementation of technology in stroke care. First, a pre/post study in Brazil examined the effects of implementing a technology based “hub (one tertiary care centre) and spoke (five emergency departments)” model for improving the frequency and accuracy of stroke diagnosis and the quality of stroke care [15]. Neurologists from the hub facility delivered training to the emergency department staff in spoke facilities, through brief lectures and live simulations that focused on providing stroke care in teams. The hub and spoke facilities were connected by a telemedicine technology that connected hub facility neurologists remotely to spoke facility desktops, to aid in the assessment of radiological images. After education and implementation of the technology, the mean number of patients diagnosed with stroke increased from 7.5 to 16.6 per month (*p* = 0.019). There was no significant change in the proportion of patients who received intravenous thrombolysis (TBL), which reduced from 21.3% to 18.2% (*p* = 0.598). There was also a decrease in symptomatic intracranial hemorrhage rate from 12.5% to 7.7% (*p* = 0.678), although not statistically significant. Mean door-to-needle time for delivery of thrombolytic agents was reduced from 137.1 to 95.5 min (*p* = 0.001).

Secondly, a 2014 retrospective case series study of a WhatsApp-based “hub (2 tertiary facilities) and spoke (17 government district hospitals with no neurological service)” model in India examined the number of patients provided with TBL [20]. Medical officers from district hospitals (medicine graduates and internal medicine postgraduates) attended workshops on stroke recognition, the use of CT scans, and WhatsApp use to connect with the hub facility where 4 neurologists were located. They were also given written protocols on the use of thrombolysis in ischemic stroke. It was unclear if the neurologists provided the training. Between June 2014 and May 2015, 26 patients received tissue plasminogen activator (TPA), which was a 100% increase since no patients were previously receiving this therapy. The number of TBL-eligible patients before and after the intervention’s implementation was not provided.

Third, researchers in China conducted a 1-year, cluster randomized controlled trial to assess the feasibility of a primary care-based mobile health app to improve stroke management in rural China (part of the SINEMA intervention) [16]. Researchers gave village doctors an android phone with the app installed and trained the doctors on how to use the app. The application used evidence-based care content and included videos, graphs, texts, and a list of important medications to assist village doctors’ decision making on medication prescription (patient outcomes were not reported in this design, development and feasibility publication). The evidence-based care content was developed from the Chinese Clinical Guideline for Secondary Prevention in Stroke in Primary Healthcare Setting [22]. The study was conducted in 50 villages, of which 25 were in the intervention group. Almost all the village doctors in the intervention group (*n* = 24, 96%) responded to a 12-month post-trial survey on the feasibility of the mHealth app. All respondents agreed or strongly agreed that the app was quick and easy to learn and supported decision making for medication prescription. Furthermore, 92% agreed or strongly agreed that they would continue using the app after the trial.

### On-site workshops for education on task-shifting to develop knowledge on stroke for non-specialists

One survey study from Nigeria examined the use of task-shifting education for developing knowledge on stroke and its care [21]. Task-shifting was defined as a strategy to enable more efficient use of available human resources whereby a task normally performed by a physician is transferred to another health professional with different or lower training or to a person trained specifically for a limited task only. A one-day workshop delivered by the research team (professions unknown) provided basic information on stroke risk factors, symptoms, development, diagnosis, and treatment to medical officers, nurses, and community health extension workers without any specialty neurological training. The education was delivered with didactic presentations, an educational stroke documentary film, and practical/interactive sessions on cases. Pre- and post-workshop self-administered stroke literacy tests were done, of which 55.2% and 91.0% were completed, respectively. Significant improvements in knowledge were noted across a variety of topics including knowledge of risk factors (90.5% vs. 99.0%, *p* < 0.001), stroke symptoms identification (79.3% vs. 90.6%, *p* < 0.001), the development of stroke (81.0% vs. 95.3%, *p* = 0.009), swallowing tests (61.2% vs. 86.9%, *p* < 0.001) and appropriate use of thrombolytics (62.9% vs. 85.9%, *p* = 0.002).

### Training the trainer for the implementation of multidisciplinary stroke units

An Indian pre/post implementation study used a “train-the-trainer” approach to implement a stroke unit (SU) [17]. The trainer (one physician) was trained by local experts for two hours via video conference (Skype), three times a week for four weeks. The trainer then conducted two-hour training sessions with healthcare professionals in nursing, physiotherapy, and occupational therapy for five days per week over four weeks. After this training, the multidisciplinary team continued to meet weekly to discuss patient treatment. All training was augmented with the use of written care protocols. After the implementation of the stroke unit by the healthcare team (after training), improvements were noted in the number of swallowing assessments (0% vs. 36.8%, *p* < 0.001), mobility assessments and patient re-education for daily tasks (91.2% vs. 99.2%, *p* = 0.037). The overall incidence of medical complications was significantly reduced (44.8% vs. 28.0%, *p* < 0.006), as was length of stay (5 ± 2.68 vs. 4 ± 2.16 days, *p* < 0.026). There was no significant difference in functional outcome at one-month between the groups, as recorded by the modified Rankin scale (good outcome 35.7% pre-SU vs. 39.6% post-SU, bad outcome 64.3% pre-SU vs. 60.4% post-SU, *p* = 0.552).

A study from Ghana described the use of a “train-the trainer” approach supported by healthcare professionals in the United Kingdom to implement a multidisciplinary stroke unit, in a program called the “Wessex-Ghana Stroke Partnership” [14]. A multi-disciplinary team of health professionals working in stroke care from the United Kingdom helped to provide the initial training to trainers (champions) and develop them into leaders to oversee different health services within a hospital setting. The train-the-train approach was supported with teaching materials and a practical competency framework, which sought to ensure that clinical skills were translated into practice. Overall, education seemed to have positive effects on the intended outcome of developing an effective multidisciplinary stroke unit. Deputy trainers and link individuals that connected specialties in stroke care together were also developed. These individuals allowed for key skill areas in stroke care to develop, with deputy trainers being implemented with succession planning in mind. An emphasis was also placed on dismantling tendencies of units that were restrictive towards collaboration between hospital services, so that multidisciplinary care approaches could be developed amongst the healthcare team.

### Training the trainer for quality improvement

The “train-the-trainer” model was also used in a Chinese multicentre, cluster-randomized clinical trial to improve acute ischemic stroke (AIS) care with 2400 patients each in the intervention and control groups [18]. The director of the neurology department and a nurse or physician from the 20 hospitals in the intervention group attended a two-day workshop on operational methods for stroke care (profession of the trainers unknown), and then shared the information they learned with other professionals from their site (complemented by training videos or slides). A physician or nurse acted as a quality coordinator at each intervention site and were responsible for staff training, identifying gaps in intervention adherence and identifying barriers to implementation. This was supported by written clinical pathways and protocols and a monitoring and feedback system. Patients in the intervention group were more likely to receive at least one of the composite performance measures (88.2% vs. 84.8%, *p* = 0.003, 95% CI 0.68% to 6.40%, adjusted for hospital and patient baseline characteristics), but not all of the performance measures (53.8% vs. 47.8%, *p* = 0.06). New clinical vascular events were lower at 3 months (3.9% vs. 5.3%, *p* = 0.002), 6 months (6.3% vs. 7.8%, *p* = 0.004), and 12 months (9.1% vs. 11.8%, *p* < 0.001) for the intervention group.

### Multidimensional education for quality improvement

A cluster randomized control trial from Brazil, Argentina, and Peru [19] evaluated the effects of a multi-faceted quality improvement intervention for the treatment of patients presenting with acute ischemic stroke (AIS) and transient ischemic attack (TIA). A total of 19 sites were randomized to the intervention group (817 patients during primary analysis until discharge, 684 at 90-days follow-up). 16 hospitals were randomized to routine care (807 patients in primary analysis until discharge, 698 after 90 days follow-up). The intervention included case management (physician lead and trained nurses from each hospital), reminders, roadmaps, checklists, educational materials and audit and feedback reports. For training, hospitals in the intervention arm received onsite training supplemented by telephone and web-based training. Further, two health care professionals from each hospital attended a workshop on the intervention implementation. Training was provided by the quality improvement team of the BRIDGE stroke investigators (further details on the trainers not provided). Analyses were adjusted for the cluster design and the presence of a stroke unit. Eligible patients in the intervention clusters received more prescriptions for evidence-based therapies than those in control clusters (73.5% vs. 58.7%). There were no significant differences between the intervention and control groups in secondary prevention efforts such as pharmaceuticals for hyperlipidemia, hypertension, or diabetes. The intervention had no effect on stroke reoccurrence (1.3% vs. 0.6%, *p* = 0.13), total mortality (12.6% vs. 11.8%, *p* = 0.58), and cardiovascular mortality (2.1% vs. 1.7%, *p* = 0.42). There were more hemorrhagic transformations identified in the intervention group compared to the control group (5.1% vs. 2.5%, *p* = 0.02).

## Discussion

We conducted a systematic review of studies examining stroke care education strategies for hospital-based healthcare professionals in low-resource settings, to better understand the reported impacts of these interventions on knowledge, behaviours and outcomes in these environments.

A train-the-trainer approach for the implementation of multidisciplinary stroke units was the most used method for education and found to be effective in two studies examined in this review [14, 18]. Both improvements in patient care and professional skills were evident. External to this review, expert opinion provided in other articles regards multidisciplinary SUs to be a gold standard in stroke care with numerous benefits [4, 23, 24]. A key strength of the “train-the-trainer” approach is potential sustainability – the Indian study by John et al. (2021) [17] and Wessex-Ghana [14] study by Johnson et al. (2017) demonstrated that healthcare professionals can educate their co-workers upon training, and the Wessex-Ghana Stroke Partnership has appointed deputies to support clinical leaders with the aim of succession planning. Whilst a train-the-trainer approach may be effective and implemented with existing human resources, successful approaches, such as in the Wessex-Ghana Stroke Partnership, have involved significant input from external healthcare expert professionals. It is important to note that educational interventions with international partnerships that require the “train-the-trainer” approach may be time consuming – the intervention in India took two months, while the Wessex-Ghana Stroke Partnership took several years. The Wessex-Ghana study also required international professionals from the United Kingdom and grant funding to augment local expertise. As local expertise grows, the need for international collaboration will likely decrease, with the added benefit of interventions becoming more tailored to the local context in the deliverance of education.

Locally-created training videos on the Wessex-Ghana website are freely available [25]. Other resources, such as the Core Competencies e-learning resource from the Stroke Training and Awareness Resources (STARs) project, are also available and accessible to learners with a varying knowledge-base [26]. However, educational resources and guidelines from the STARs project are from western high-resource settings, and as such may not always be relevant for global low-resource settings. More research is needed to determine if these resources and guidelines, or components of them, can be applicable to low-resource settings.

Of the care quality improvement initiatives, the study in China by Wang et al. (2018) with the “train-the-trainer” model had the most demonstrated potential [18]. The delivery of performance measures, when they were assessed as a composite, was increased and the occurrences of new clinical vascular events at 3, 6, and 12 months was decreased. In comparison, the South American study by Machline-Carrion et al. (2019) which also sought to implement overall care quality improvement had increased prescriptions for evidence-based therapies, but no significant differences in secondary prevention efforts, stroke reoccurrence, and mortality measures [19]. Furthermore, the number of hemorrhagic transformations was increased, but it is unclear if this was due to improved detection, better recording of clinical notes, and/or confounding variables.

Three studies [15, 16, 20] demonstrated that technology can be used to improve patient care in low-resource settings. A telemedicine model with advanced computing and imaging facilities like the one used in Brazil [15] by Carvalho et al. (2019) may be out of reach for some given the diversity of facilities in different regions. The Indian initiative by Sharma et al. (2016) used WhatsApp, a readily accessible and freely available mobile messaging application with end-to-end encryption [20, 27]. A study examining the perceptions of healthcare workers on how to improve the quality of stroke care in Ghana noted a recommendation for the use of technological tools like WhatsApp, which was thought to have the ability to promote collaborative or multidisciplinary care [8]. In the Chinese study by Wu et al. (2019), village doctors and patients used a smartphone application for both communication and the provision of education [16]. The overall reception of the implementation of smartphones was positive among village doctors in this study and it was perceived to improve their ability to provide healthcare. However, building a new application like in the Wu et al. (2019) study may not be feasible or necessary for some and therefore the use of WhatsApp or similar free messaging apps should be strongly considered. Finally, Wu et al. (2019) study noted that they chose android smartphones because they were relatively affordable and popular for their setting. The use of applications on android smartphones may be the most feasible avenue for delivering education on stroke diagnosis through technology. Technology use in stroke education may also augment the delivery of other educational models, such as the “train the trainer” approach by John et al. (2021) [17] and the multidimensional approach by Machline-Carrion et al. (2019) [19]. However, in areas with limited connectivity to internet and cellular services, the use of technology may not be feasible until local infrastructure changes are made.

On-site workshops were used in Nigeria in a study by Akinyemi et al. (2015) to provide non-specialists with knowledge on stroke and its care [21]. Data were not provided on whether this approach improved patient care or outcomes, but the education of non-neurologist healthcare workers on stroke and patient care was demonstrated through literacy questionnaires. This indicates that simple strategies for sharing basic knowledge on stroke care in settings where there are no specialists may have potential in improving outcomes [5, 6]. In contrast, multidimensional educational training for nurses and physicians in Brazil, Argentina, and Peru by Machline-Carrion et al. (2019) had a variable effect on patient outcomes, suggesting that complex education using elements of multiple educational themes may not be better [19].

Although data are limited, it seemed that the “train the trainer” approach was most optimal in providing stroke care education. Technology can be useful as well and if resources are limited, basic education should be considered at a minimum. Previous surveys have shown that health professionals in low-resource settings consider education to be helpful and prefer to do so in workshop and conference formats [8, 28, 29]. Many of our examples included external trainers, however these findings indicate interest in learning with others in the same settings. The interest in learning in a group could be extrapolated to the creation of communities of practice that have been shown to be effective in sustaining educational efforts and facilitating implementation of knowledge [30, 31]. An approach that may be helpful is the establishment of communities of practice so that local contexts can be considered in the design of educational interventions. The African Stroke Organization (ASO) is an example of a community of practice for stroke care professionals [32]. The ASO is composed of stroke researchers, clinicians, allied healthcare professionals, stroke care societies, and stroke support organizations in Africa. Annual conferences are planned in which knowledge gaps on stroke are addressed, research is enhanced, collaboration between African regions is increased, priorities for care delivery are guided, and leaders are built. The Organized Stroke Care Across Income Levels (OSCAIL) study is another example of a community of practice, with healthcare professionals from high and low-resource settings collaborating with an aim to improve post-stroke outcomes through simple evidence-based strategies [33].

While our review is comprehensive, there are some major limitations. Firstly, we could not meta analyse results as the studies were too heterogenous in their methodologies and measured outcomes. Secondly, some studies did not evaluate the impact of training interventions directly on patients. Thirdly, there was no clear definition of what constitutes an effective intervention. Fourthly, we were unable to determine if the changes observed in individual studies were due to Hawthorne effects, improvements in data capture over time, or other biases. Fifthly, multiple educational strategies were used in some studies, making it difficult to clearly identify the individual impacts of the educational components. Several of the studies included written care protocols, pathways and road maps along with educational components. It is possible that the provision of these protocols alone would have resulted in the same positive impact on care. Finally, only 8 out of 1,182 articles from our search met inclusion criteria. The majority of articles were excluded for three reasons: did not describe educational approaches (~ 40%), were not specific to stroke training and did not occur in a low-resource setting. The large number excluded could be due to non-specific search criteria which were required due to the lack of systematic indexing and the use of a variety of key words.

The quality of evidence of the included articles was generally satisfactory. Randomized control trial methods in future studies can reduce the risk of biases and increase the level of confidence in the results. While randomized control trials remain the gold standard for evaluating effectiveness, they are not the only design nor is it always the most appropriate design (e.g., sometimes it is difficult to randomize individuals in a setting and a cluster design is not feasible). Other robust study designs, such as cohort or case–control studies, can also be considered. These studies could be implemented at an institutional level where one institution provides training and then implementation outcomes are studied (cohort) or different institutions in the same context (geographical area for example) participate with one receiving training and the other acting as a control (case–control). A pragmatic approach to trial design should be considered, that is informed by a multidisciplinary team and with patient input.

## Conclusions

This systematic review demonstrates that different educational interventions have the potential to deliver positive results for speciality stroke care education in low-resource settings for hospital-based healthcare professionals. A “train-the-trainer” approach to education was deemed to have the most potential, with technology use also being promising. Regardless of resources, basic education for the development of knowledge on stroke and its care should be considered as a minimum requirement. Multidimensional approaches that implement elements from multiple educational themes for care improvement may not have as much potential as other approaches. We ultimately recommend further research that focuses on designing educational interventions within communities of practice, so that the impacts of local contexts in design may be considered.

## Supplementary Information

**Additional file 1.**

## Data Availability

Database search strings are available from the corresponding author on reasonable request.

## Abbreviations

PRISMA: Preferred Reporting Items for Systematic Reviews and Meta-Analyses
TBL: Intravenous thrombolysis
TPA: Tissue plasminogen activator
SU: Stoke unit
STARs: Stroke Training and Awareness Resources
AIS: Acute ischemic stroke
TIA: Transient ischemic attack
