# Supplementary material for: Strategies for specialty training of healthcare professionals in low-resource settings: a systematic review on evidence from stroke care
Source: BMC Med Educ. 2023 Jun 16;23:442. doi: 10.1186/s12909-023-04431-w (PMC10273731; doi:10.1186/s12909-023-04431-w)
Supplement: Supplementary file 1 — Additional file 1. [file 12909_2023_4431_MOESM1_ESM.docx]

**SEARCH: #1 AND #2 AND #3 AND #4**

**(#1)** “professional*” OR “attitude of health personnel*” OR “health personnel” OR “health

worker*” OR “staff” OR “healthcare personnel” OR “healthcare worker*” OR “caregiver*” OR

“carer*”

**(#2)** “education*” OR “survey*” OR “questionnaire*” OR “training*” OR “teaching*” OR

“workshop*” OR “professional development*”

**(#3)** “stroke*”

**(#4)** “developing countr*” OR “developing population*” OR “developing world” OR

“developing econom*” OR “underdeveloped countr*” OR “underdeveloped population*” OR

“underdeveloped world” OR “underserved countr*” OR “underserved population*”

“underdeveloped econom*” OR “least developed econom*” OR “less developed nation*” OR

“less developed countr*” OR “less developed population*” OR “less developed world” OR

“least developed population*” OR “least developed countr*” OR “least developed nation*” OR

“low income countr*” OR “lower income countr*” OR “lowest income countr*” OR “low

income nation*” OR “lower income nation*” OR “lowest income nation*” OR “low income

population*” OR “lower income population*” OR “lowest income population*” OR “low and

middle income” OR “middle income countr*” OR “middle income population*” OR “middle

income nation*” OR “middle income econom*” OR “poor countr*” OR “poorer countr*” OR

“poorest countr*” OR “poor nation*” OR “poorer nation*” OR “poorest nation*” OR “poor

population*” OR “poorer population*” OR “poorest population*” OR “poor world” OR “third

world” OR “transitional countr*” OR “transitional econom*” OR “transitional population*” OR

“low gdp” OR “low gnp” OR “low gross domestic” OR “low gross national” OR “lmic*” OR

“lami countr*” OR “low resource*”

**MeSH Terms Used:**

• Attitude of Health Personnel*

• Evidence-Based Practice / methods*

• Health Personnel / statistics & numerical data

• Humans

• Qualitative Research

• Quality of Health Care / statistics & numerical data*

• Stroke / therapy*

• Surveys and Questionnaires
